# Heart Rate Monitoring Among Breast Cancer Survivors: Quantitative Study of Device Agreement in a Community-Based Exercise Program

**DOI:** 10.2196/51210

**Published:** 2024-06-20

**Authors:** Lindsey L Page, Jason Fanning, Connor Phipps, Ann Berger, Elizabeth Reed, Diane Ehlers

**Affiliations:** 1 Department of Neurological Sciences University of Nebraska Medical Center Omaha, NE United States; 2 Department of Health and Exercise Science Wake Forest University Winston-Salem, NC United States; 3 College of Nursing University of Nebraska Medical Center Omaha, NE United States; 4 Department of Internal Medicine University of Nebraska Medical Center Omaha, NE United States; 5 Department of Quantitative Health Sciences Division of Epidemiology Mayo Clinic Arizona Rochester, AZ United States

**Keywords:** wearable devices, exercise prescription, validity, photoplethysmography, monitoring, wearables, devices, exercise, heart rate, breast cancer, cancer, cancer survivor, community, chest monitor, Fitbit, recovery, safety

## Abstract

**Background:**

Exercise intensity (eg, target heart rate [HR]) is a fundamental component of exercise prescription to elicit health benefits in cancer survivors. Despite the validity of chest-worn monitors, their feasibility in community and unsupervised exercise settings may be challenging. As wearable technology continues to improve, consumer-based wearable sensors may represent an accessible alternative to traditional monitoring, offering additional advantages.

**Objective:**

The purpose of this study was to examine the agreement between the Polar H10 chest monitor and Fitbit Inspire HR for HR measurement in breast cancer survivors enrolled in the intervention arm of a randomized, pilot exercise trial.

**Methods:**

Participants included breast cancer survivors (N=14; aged 38-72 years) randomized to a 12-week aerobic exercise program. This program consisted of three 60-minute, moderate-intensity walking sessions per week, either in small groups or one-on-one, facilitated by a certified exercise physiologist and held at local community fitness centers. As originally designed, the exercise prescription included 36 supervised sessions at a fitness center. However, due to the COVID-19 pandemic, the number of supervised sessions varied depending on whether participants enrolled before or after March 2020. During each exercise session, HR (in beats per minute) was concurrently measured via a Polar H10 chest monitor and a wrist-worn Fitbit Inspire HR at 5 stages: pre-exercise rest; midpoint of warm-up; midpoint of exercise session; midpoint of cool-down; and postexercise recovery. The exercise physiologist recorded the participant’s HR from each device at the midpoint of each stage. HR agreement between the Polar H10 and Fitbit Inspire HR was assessed using Lin concordance correlation coefficient (*r*_c_) with a 95% CI. Lin rc ranges from 0 to 1.00, with 0 indicating no concordance and 1.00 indicating perfect concordance. Relative error rates were calculated to examine differences across exercise session stages.

**Results:**

Data were available for 200 supervised sessions across the sample (session per participant: mean 13.33, SD 13.7). By exercise session stage, agreement between the Polar H10 monitor and the Fitbit was highest during pre-exercise seated rest (rc=0.76, 95% CI 0.70-0.81) and postexercise seated recovery (rc=0.89, 95% CI 0.86-0.92), followed by the midpoint of exercise (rc=0.63, 95% CI 0.55-0.70) and cool-down (rc=0.68, 95% CI 0.60-0.74). The agreement was lowest during warm-up (rc=0.39, 95% CI 0.27-0.49). Relative error rates ranged from –3.91% to 3.09% and were greatest during warm-up (relative error rate: mean –3.91, SD 11.92%).

**Conclusions:**

The Fitbit overestimated HR during peak exercise intensity, posing risks for overexercising, which may not be safe for breast cancer survivors’ fitness levels. While the Fitbit Inspire HR may be used to estimate exercise HR, precautions are needed when considering participant safety and data interpretation.

**Trial Registration:**

Clinicaltrials.gov NCT03980626; https://clinicaltrials.gov/study/NCT03980626?term=NCT03980626&rank=1

## Introduction

Wearable sensors have gained traction in both commercial and research sectors [1], with a projected 156 million units to be purchased in 2024 [2]. These devices use microelectronic triaxial accelerometers to measure steps, energy expenditure, sleep, and time spent in different intensities of activity and photoplethysmography above the wrist to measure heart rate (HR). These data, along with options for goal setting, can be used to help individuals self-monitor and increase their daily physical activity (PA) [3]. The ease and utility of these devices have led to their adoption in health promotion research for continuous measurement of health behaviors and as behavior change tools [4].

Traditional research-grade monitors are costly, lack consumer-friendly designs, provide little opportunity for user interaction with the device, often evaluate only 1 dimension of daily activities (eg, HR, motion, or sleep only), and have limited, real-time data transfer capacity [3,5,6]. In contrast, consumer-grade monitors continuously collect and transfer data through Bluetooth and web-based platforms to allow for data collection across months or even years [3]. Additionally, participant data can be easily monitored and accessed via web-based platforms at any point during the data collection period. The increasing number of peer-reviewed publications and National Institutes of Health–funded grant proposals, which include consumer-grade, wrist-worn monitors, emphasizes the utility of these devices in research settings [1,7].

Many of these devices now measure HR, a key component of aerobic exercise prescription. Although electrocardiogram (ECG) is widely accepted as the gold standard for assessing HR during exercise, chest-worn monitors also have well-documented validity for measuring HR [8]. However, like ECG, they may be inconvenient or prohibitive in community-based and unsupervised exercise settings due to necessary receivers and participant discomfort. In contrast, newer devices are increasingly being designed for wear on the forearm or wrist. Commercially available wearable sensors, such as the Fitbit, represent an accessible, multifunctional alternative to HR monitoring in exercise. Appropriate exercise intensity, often expressed as a percentage of HR reserve, is a fundamental dimension of exercise prescription for achieving the health benefits of exercise [9]. For example, cancer survivors begin to reduce fatigue symptoms with a minimum dose of aerobic activity at 45% of their HR reserve, whereas benefits for other symptoms (ie, anxiety, depression, and physical function) begin at a minimum dose of 60% of their HR reserve [10]. To improve dissemination and uptake of exercise prescriptions in clinical or community-based settings, it is critical that survivors have user-friendly methods to independently monitor exercise prescription components.

There is an increasing number of exercise oncology studies that use commercial wearable sensors to intervene in PA behaviors and reduce cancer-related symptom burden, particularly among breast cancer survivors, and evidence indicates that wearable sensors are effective, feasible, and user-friendly for breast cancer survivors in exercise interventions [11-13]. Although these studies have helped bolster the utility of wearable sensors in PA promotion research, they have failed to provide any detail on intensity or HR monitoring during their respective interventions. Many exercise interventions in cancer populations are adopting community-based, hybrid, and unsupervised designs [14]. Therefore, it is integral that researchers understand the capacity, and limitations, of commercially available wearable sensors in providing accurate measurements of HR to monitor participants’ safety and compliance with the exercise prescription.

In the general population, the reliability of popular, commercially available activity and HR monitors has been previously examined with varying agreement between commercial products and traditional ECG monitoring [8,15-17]. Unfortunately, many of these data have been collected in controlled settings with predetermined treadmill speeds in young, healthy adult participants. The dearth of literature assessing commercially available, wrist-worn HR monitors in clinical populations during training sessions limits the utility of these devices in less controlled environments. Therefore, the purpose of this study was to examine the agreement between a commercially available, wrist-worn wearable sensor (Fitbit Inspire HR; Fitbit Inc) and a traditional chest-worn monitor (Polar H10; Polar Electro OY) for HR measurement in breast cancer survivors at different stages of exercise in a community-based program. It is hypothesized that HR monitor agreement in this study will be highest at periods of pre- and postexercise rest and lowest during the exercise session when participants were exercising at higher intensities.

## Methods

### Study Design and Participants

The Study on Physical Activity’s Relationship with Cancer and Cognition (SPARCC) was a randomized exercise trial in which 30 women diagnosed with breast cancer were randomized to a 12-week moderate-intensity aerobic exercise program (n=15) or usual care (n=15). Our study includes only those women randomized to the exercise group with valid Fitbit and Polar HR data (n=14), as exercise HR was not collected from women in the usual care control group.

Eligibility criteria for this study included the following: female participants aged 21 years or older; postmenopausal at the time of diagnosis; first, primary diagnosis of breast cancer (stage I-IIIa); within 3-24 months of completing surgery, chemotherapy, or radiation therapy; self-reported an average of <60 minutes of moderate to vigorous PA per week for the previous 6 months; having received physician’s clearance to participate in an exercise program; and randomized to the 12-week aerobic exercise program in the SPARCC study. Participants were recruited from a midwestern academic medical center, a private cancer center, and the community (eg, via flyers to community organizations, social media posts, and word of mouth). Interested individuals were scheduled for a phone appointment to confirm eligibility, absence of neurological disorders, and interest in participating in the study. Eligible women were then asked to attend an in-person or Zoom-based orientation session to receive more information about the study, decide if they would like to participate, sign the Institutional Review Board (IRB)–approved informed consent, and schedule baseline testing appointments. After baseline data collection was complete, participants were randomized in a block design to the 12-week aerobic exercise program or usual care. Participants were not instructed to change physical activity behaviors prior to beginning the exercise program.

### Ethical Considerations

This study was approved by The University of Nebraska Medical Center IRB and is registered with the National Institutes of Health’s ClinicalTrials.gov (NCT03980626). All participants provided written informed consent prior to participation. All data presented herein were deidentified using study identification numbers and stored separately from participants. identifiers. Data were collected and managed using applications hosted by the study institution (ie, Research Electronic Data Capture [REDCap] or Box Enterprise) [18,19]. Participants did not receive payment for their participation in this research but received a Fitbit Inspire HR that was theirs to keep. All participants were offered a 3-month membership to a local fitness center.

### Exercise Protocol

Breast cancer survivors randomized to the exercise program engaged in small group or one-on-one, moderate-intensity walking sessions facilitated by a certified exercise physiologist. These sessions were held at local community fitness centers 3 times per week for 1 hour per session. All participants completed a treadmill-based submaximal cardiopulmonary exercise test prior to randomization to establish baseline fitness and safety and inform individualized exercise prescriptions (Table 1).

**Table 1:** Exercise prescription.

Week	Intensity (rating of perceived exertion), range	Intensity (% heart rate reserve), range	Duration (minutes), range
1	9-11	45-50	15-20
2	9-11	45-50	20-25
3	9-11	45-50	25-30
4	11-13	50-55	25-30
5	11-13	50-55	30-35
6	11-13	50-55	35-45
7	13-15	55-65	35-45
8	13-15	55-65	40-50
9	13-15	55-65	40-50
10	15-17	65-75	40-50
11	15-17	65-75	45-50
12	15-17	65-75	45-50

As originally designed, all 36 exercise sessions were scheduled to be delivered by the exercise physiologist in the supervised, community-based setting. However, due to the COVID-19 pandemic, the exercise program was modified for some participants to include both supervised and unsupervised sessions. Participants who were in the middle of the intervention in March 2020, were transitioned to unsupervised exercise with weekly Zoom-based counseling from their trainer. Participants enrolled after March 2020, engaged in only 4 supervised exercise sessions held once per week in the research team’s exercise laboratory in weeks 1-4. All sessions in weeks 7-12 were unsupervised, home-based sessions with weekly Zoom-based exercise counseling. Across the study, 4 breast cancer survivors completed 36 supervised sessions as originally designed, 5 were in the middle of the intervention in March 2020, and 6 were enrolled after March 2020 and received 4 supervised sessions. Depending on the enrollment time (ie, before or after the COVID-19 public health restrictions), participants engaged in an average of 13.33 (SD 13.71) supervised sessions (range: 4-36). Participants who received the original intervention received their fitness center membership during the study. Those enrolled during or after the onset of the COVID-19 pandemic were offered a fitness center membership when it was safe to do so based on local IRB and public health requirements. Only supervised sessions (n=200) were included in our analysis.

**Figure 1:**
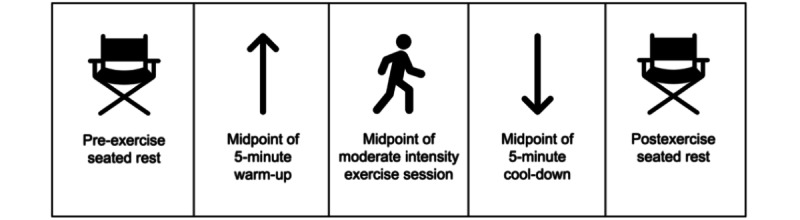
Exercise session stages for heart rate measurement.

The exercise program was progressive in nature such that the volume of exercise increased across weeks from 15-20 minutes of walking in weeks 1-2 to 40-45 minutes in weeks 8-12 and from 40%-55% estimated HR reserve in week 1 to 65%-70% HR reserve in weeks 9-12 (Table 1). All sessions began with a 5-minute light-intensity walking warm-up and ended with an active cool-down including light walking and static stretches (Figure 1). The exercise program was designed to follow American College of Sports Medicine guidelines for exercise in cancer survivors [10].

### Measures

#### HR Monitors

All participants received a Fitbit Inspire HR sensor to wear on their nondominant wrist for the duration of the study and a Polar H10 chest strap to wear during supervised exercise. The Fitbit was chosen for this study because it is one of the most popular wrist-worn activity trackers, represents approximately 20% of the commercial wearable sensor market, and has sold 63 million devices worldwide in the last decade [3]. The Fitbit Inspire HR measures HR via optical photoplethysmography, which is processed using proprietary algorithms. Briefly, this is done by shining a light on the skin, assessing the reflected light, using algorithms to determine changes in blood volume based upon reflected light, and calculating HR based on oscillations in blood volume [20,21]. The Polar H10 chest strap monitor was chosen as the comparator device because it has high validity with ECG, the gold standard for measuring exercise HR [8]. Exercise trainers fit participants with the Polar H10 monitors, placed on the distal sternum, at the start of each supervised exercise session and used Polar HR readings to adjust treadmill speed and grade to meet prescribed exercise intensity. HR was measured concurrently using the Fitbit and Polar monitors at 5 stages of the exercise session: pre-exercise seated rest; midpoint of the 5-minute warm-up; midpoint of the moderate-intensity exercise; midpoint of the 5-minute cool-down; and after a 5-minute seated recovery (Figure 1).

#### Demographic and Clinical Information

Participant demographics (ie, age, race, education, income, employment status, marital status, and comorbid conditions) were self-reported and collected via REDCap hosted by the study institution. Clinical information on breast cancer diagnosis and treatment were obtained via electronic medical records. BMI was calculated from height and weight measured via the Seca 703 scale and stadiometer (Seca Corp) by the study staff at a baseline testing visit.

### Data Analysis

HR from the Polar monitor was operationalized as the criterion measure and used to assess absolute and relative paired differences between monitors [8]. Agreement between HR measurements was assessed using Lin concordance correlation coefficient (*r*_c_) with 95% CIs. This test measures the degree to which the paired observations fall on the identity line and defines statistical agreement as *r*_c_≥0.85 [22]. The agreement was also represented visually across stages using Bland-Altman plots with upper and lower limits set using 95% CIs [23,24]. Absolute paired differences were calculated by subtracting the Fitbit-measured HR from the Polar-measured HR at each stage of the exercise session. Relative paired differences were calculated as relative error rate (RER) across exercise session stages [25,26], as follows:

RER = (Polar HR measurement – Fitbit HR measurement) × 100/Polar HR

Negative resultant RERs are indicative of an overestimation of HR by the Fitbit, and positive RERs are indicative of an underestimation of HR by the Fitbit, as compared to the Polar monitor. Data were analyzed using SPSS (version 27; IBM Corp) and RStudio (version 1.3.1093; R Core Team).

## Results

### Participant Characteristics

Participants (mean age 63.1, SD 8.7 years) were White women with a history of early-stage breast cancer; on average, overweight; and physically inactive (Table 2). Additionally, more than 1 quarter of participants had a history of clinically diagnosed anxiety or depression at the time of enrollment. Breast cancer survivors in this study were enrolled approximately 17 months after their diagnosis. Participants’ breast cancer treatments included surgery, radiation, and chemotherapy; however, most women in this study did not receive chemotherapy. One participant randomized to the exercise program did not have valid Polar data for supervised sessions and was, therefore, excluded from the analysis (N=14).

**Table 2:** Participant characteristics.

Characteristics				Values
**Demographics**				
	Age (years), mean (SD)		63.07 (8.66)	
	Non-Hispanic White, n (%)		13 (93)	
	Bachelor’s degree, n (%)		8 (57)	
	Income >US $40,000 per year, n (%)		8 (57)	
	Employed full-time, n (%)		8 (57)	
	Married, n (%)		12 (86)	
	Comorbidities^a^, mean (SD)		2.38 (2.10)	
	β-Blocker medication use, n (%)		0 (0)	
	Antihypertensive medication use, n (%)		1 (7)	
	Diagnosed with depression, n (%)		4 (29)	
	Diagnosed with anxiety, n (%)		4 (29)	
	BMI (kg/m^2^), mean (SD)		29.14 (4.71)	
**Clinical features**				
	**Cancer stage, n (%)**			
		I	12 (86)	
		II	2 (14)	
	Time since diagnosis (months), mean (SD)		16.57 (7.97)	
	Chemotherapy, n (%)		3 (21)	
	Radiation, n (%)		9 (64)	
	Hormonal therapy, n (%)		9 (64)	
	Months of hormonal therapy, mean (SD)		13.44 (5.64)	

^a^Comorbid conditions include diagnosed arthritis, osteoporosis, asthma, chronic obstructive pulmonary disease, angina, heart failure, previous myocardial infarction, vascular disease, diabetes, tremors, gastrointestinal disease, visual impairment, hearing impairment, degenerative disk disease, anxiety, and depression.

### HR Monitor Agreement

**Figure 2:**
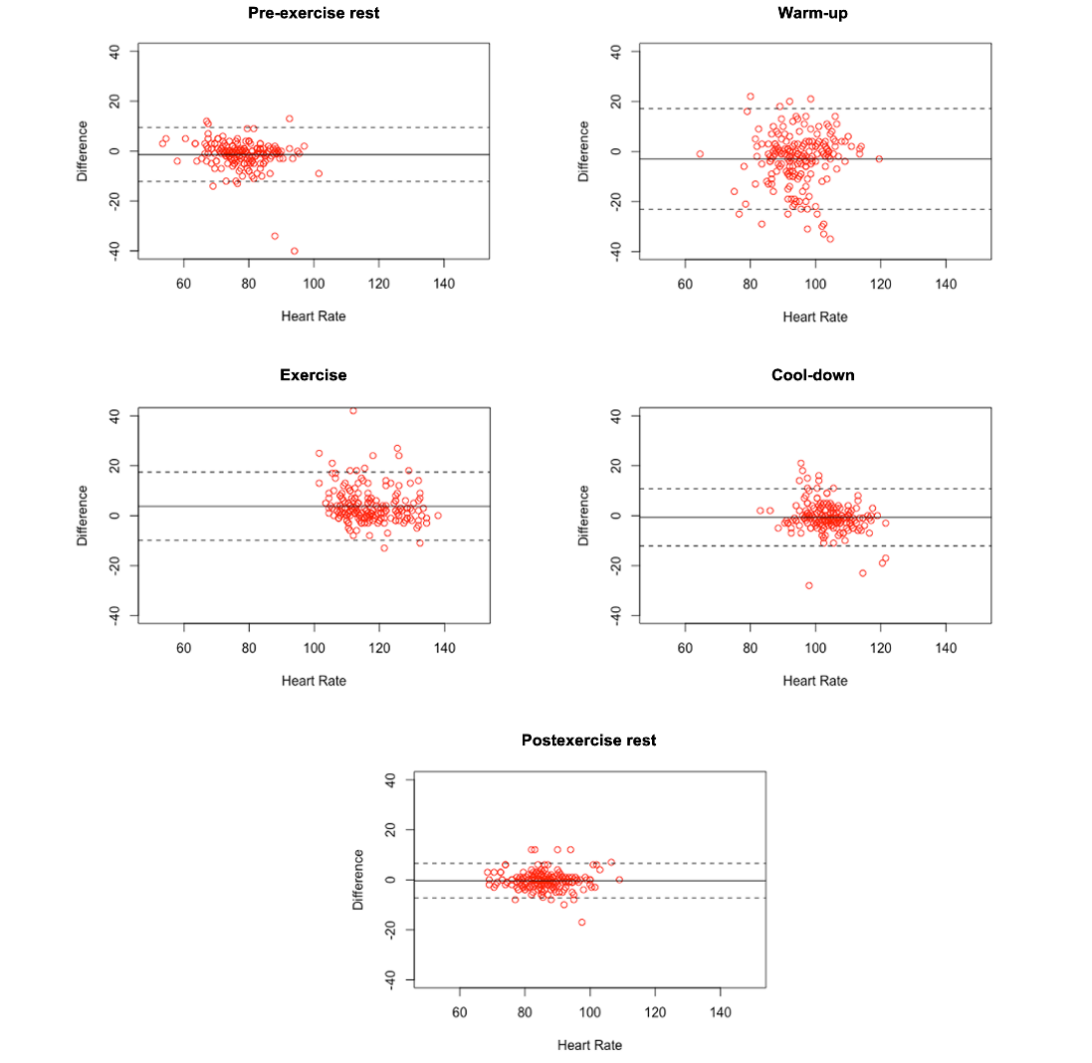
Bland-Altman plots. Bland-Altman plots depict the average heart rate (Polar H10 and Fitbit) by the relative difference between the two measures for each session by stage in the exercise protocol. Points on the plots indicate individual sessions, solid lines indicate the mean difference across the sample, and dashed lines indicate upper and lower bounds for each stage.

Agreement between the Fitbit and Polar HR monitors was highest during seated rest at postexercise (*r*_c_=0.89, 95% CI 0.86-0.92) and pre-exercise (*r*_c_=0.76, 95% CI 0.70-0.81). This was followed by the midpoint of the moderate-intensity exercise session (*r*_c_=0.63, 95% CI 0.55-0.70) and cool-down (*r*_c_=0.68, 95% CI 0.60-0.74). The warm-up was associated with the lowest level of agreement between monitors (0.39, 95% CI 0.27-0.49). RERs ranged from –3.91% to 3.09% and were most pronounced during warm-up (RER: mean –3.91%, SD 11.92%). When inaccurate, the Fitbit overestimated HR during most stages of the exercise session (RER range: –3.91% to –0.52%), except at the midpoint of moderate-intensity exercise, where HR was underestimated (RER 3.09%). RERs and concordance coefficients are provided in Table 3, and Bland-Altman plots are provided in Figure 2.

**Table 3:** Heart rate (beats per minute) monitor differences according to the stage of the exercise.

Activity	Heart rate, mean (SD)			Fitbit differences from Polar H10, mean (SD)		
	Polar H10	Fitbit	Paired difference		Percent difference (RER^a^)	Agreement (*r*_c_)^b^
Pre-exercise rest	78.00 (7.68)	78.61 (8.74)	–1.34 (5.53)		–1.84 (7.51)	0.76
Warm-up	94.78 (7.91)	96.26 (9.17)	–2.96 (10.27)		–3.91 (11.92)	0.39
Exercise	117.04 (8.10)	115.15 (9.3)	3.77 (6.98)		3.09 (5.64)	0.63
Cool-down	103.56 (6.68)	104.24 (7.90)	–0.72 (5.83)		–0.77 (5.74)	0.68
Postexercise rest	85.86 (7.42)	85.99 (7.71)	–0.38 (3.53)		–0.52 (4.04)	0.89

^a^Relative error rate.

^b^Lin concordance correlation coefficient.

## Discussion

### Principal Results

This study was the first to examine the agreement between the wrist-worn Fitbit Inspire HR and chest-worn Polar H10 HR monitors in cancer survivors and during an exercise intervention. Major findings from this study indicate that the Fitbit monitor did not produce statistically accurate measures of HR during most exercise stages, especially the warm-up stage; however, agreement during seated rest (pre- and postexercise), midpoint of exercise, and cool-down were associated with moderate agreement between devices. Data also suggest that the Fitbit underestimated HR only during the primary, aerobic portion of the exercise session (ie, the midpoint of the exercise session), which may have serious implications for safety and compliance monitoring in exercise programs. This study extends the current literature on consumer-grade, wrist-worn HR monitors and provides data to inform future studies hoping to use consume-grade sensors to monitor safety, exercise program compliance, and longitudinal behavioral patterns in cancer survivors [8,25,27]. This is particularly important as exercise interventions become less centralized and hybrid and unsupervised approaches increase in prevalence [28-30].

### Comparison With Prior Work

The wrist-worn, Fitbit Inspire HR monitor accurately measured HR only during seated rest postexercise compared to the chest-worn Polar H10 monitor. Although pre-exercise seated rest, midpoint of moderate-intensity exercise, and cool-down also exhibited high levels of agreement, they did not reach statistical agreement as defined by Lin concordance correlation coefficient [22]. Results from previous studies have found that Fitbit devices are most accurate in measuring HR during low-intensity activities where the wrist is moving in a repetitive fashion [17]. Nevertheless, in contrast to these previous studies, warm-up represented the period of the poorest agreement. It is also unclear why the Fitbit accurately measured HR during post- but not pre-exercise seated rest; however, both pre-and postexercise seated rest reflected the highest levels of agreement with the Polar monitor, consistent with previous findings [8,16]

Although HR was highest during the midpoint of the exercise session and cool-down, these stages represented higher levels of agreement when compared to the warm-up stage. This may, in part, be due to the slower speeds at which breast cancer survivors were walking in this study, as compared to healthy, young, or middle-aged adults in other studies [8,16,17,31]. While previous research found that lower treadmill speeds showed the highest agreement, overall speeds in those studies ranged from 2 to 9 miles per hour [16,17]. In comparison, breast cancer survivors in this study did not reach speeds greater than 3.5 miles per hour. It is possible that speeds in this study were more similar to light-intensity walking in previous studies, which would align more closely with the results from this study [16,17]. This does not, however, explain the poor agreement during the warm-up and cool-down stages.

Of note, the RER at the midpoint of the exercise session indicates that the Fitbit monitor underestimated HR as compared to the Polar chest strap. Previous studies have reported similar trends in Fitbit data as compared to traditional ECG monitoring [8,16,17,31]. One study found the Fitbit Ionic to be comparable to other wrist-worn monitors and statistically accurate at rest [17], while another found that the Fitbit Blaze provided the least accurate optically measured HR [8]. In contrast to the findings presented here, these studies also found that higher-intensity activity led to decreased accuracy in HR measurement [8,17]. However, differences in Fitbit accuracy at peak exercise intensity between previous studies and data presented in this study may be due to the lower absolute intensity in this study, as both previous studies were conducted among athletes [8,17]. Given the generally lower intensity of exercise prescribed to cancer survivors, participants may not reach an exercise intensity high enough for devices to decrease in accuracy during steady-state exercise. This should, theoretically, reinforce the utility of Fitbits in cancer survivor populations.

Despite this, underestimation of exercise HR may be problematic in programs using Fitbit to monitor intensity during exercise in cancer survivors for several reasons. First, participants may be asked to increase the intensity of a session to achieve the prescribed HR range. If the Fitbit monitor underestimates HR, as it did in this study, participants who reach the prescribed HR as measured by Fitbit may be exercising at an intensity higher than that prescribed, leading to concerns regarding participant safety—particularly if the session is unsupervised. In a previous analysis of exercise prescription adherence in this sample, data indicated that participants only met prescribed intensity during supervised sessions 57.5% of the time when assessed via Fitbit HR, as compared to 92.2% when measured via Polar. However, adherence to the prescribed intensity via Fitbit was 83.2% during unsupervised sessions after the onset of the COVID-19 pandemic [32]. These data, when taken together with the findings of this study, suggest that participants may have been exercising above their prescribed HR range during unsupervised sessions. Although many breast cancer survivors may comfortably exercise at higher intensities, reliance upon consumer-grade wearable sensors only may introduce safety concerns not previously observed in more traditional, controlled exercise trials. Additionally, future studies that use Fitbit to measure the dose of exercise required to effect specific outcomes (eg, cancer-related fatigue and cognitive performance) may underestimate the required intensity of activity to elicit an effect. Although these devices may have utility in exercise oncology, it is critical that researchers and practitioners are aware of limitations that may increase the risk of adverse events or decrease methodological rigor in quantifying compliance with exercise prescriptions.

### Limitations

Although this study is one of the first to investigate HR agreement between the Fitbit Inspire HR and Polar H10 chest monitor during community-based exercise in breast cancer survivors, it is not without limitations. First, this study was performed on a small sample of breast cancer survivors. Our sample was primarily comprised of White, educated women with early-stage breast cancer, which may not be representative of the larger breast cancer population. For example, women in this study were also not on any β-Blockers, and only 1 participant reported antihypertensive medication use. Although this improves the internal validity of this study due to the lack of HR suppression, it is likely not representative of the broader breast cancer survivor population in the United States [33]. Future studies would be strengthened by the inclusion of a larger, more diverse sample of breast cancer survivors with more supervised exercise sessions.

Additionally, exercise physiologists were available to help and provide feedback on using the devices during the exercise sessions, making it unclear to what extent user error would influence Fitbit’s accuracy in unsupervised exercise settings. Fitbit HR measurements in this study were also compared to Polar chest strap monitors, rather than the gold standard ECG. This may have introduced systematic error in evaluating agreement. Finally, the total number of sessions observed in this study was fewer than originally planned (ie, 200 observed vs 540 planned) due to COVID-19 required adaptations. It is unclear whether additional observations would have changed or stabilized results relative to device agreement.

### Conclusions

Overall, Fitbit devices with HR monitoring capabilities may be useful for participant monitoring in exercise oncology studies. Researchers should use caution when using these devices, however, as they likely do not provide accurate HR measurement during critical stages of exercise sessions. This study showed that Fitbit monitors were only statistically accurate during seated rest and likely underestimated HR during steady-state exercise. As a result, Fitbit HR measurements are likely best for estimating exercise intensity rather than evaluating compliance with exercise prescriptions. Because of their ease and potential utility in behavioral PA interventions, future studies should further examine the agreement between wrist-worn wearable sensors and a gold-standard measurement of HR, such as ECG, in a larger, more representative sample of breast cancer survivors. Additionally, studies should analyze agreement by relative HR intensity to determine whether Fitbit may be more appropriate for specific exercise prescriptions.

## Abbreviations

ECG: electrocardiogram
HR: heart rate
IRB: Institutional review board
PA: physical activity
REDCap: Research Electronic Data Capture
RER: relative error rate
SPARCC: Study on Physical Activity’s Relationship with Cancer and Cognition

## Data Availability

The data sets generated during this study are available from the corresponding author on reasonable request.
